# How obesity affects nasal function in obstructive sleep apnea: anatomic and volumetric parameters^☆^

**DOI:** 10.1016/j.bjorl.2020.06.002

**Published:** 2020-07-21

**Authors:** Marcos Marques Rodrigues, Pedro Henrique de Azambuja Carvalho, Mário Francisco Real Gabrielli, Ricardo Nasser Lopes, Otávio Alves Garcia Junior, Valfrido Antonio Pereira Filho, Luis Augusto Passeri

**Affiliations:** a Universidade de Araraquara (Uniara), Faculdade de Medicina, Divisão de Otorrinolaringologia, Araraquara, SP, Brazil; b Universidade Estadual de São Paulo (UNESP), Faculdade de Odontologia de Araraquara, Departamento de Diagnóstico e Cirurgia, Araraquara, SP, Brazil; c Universidade Estadual de São Paulo (UNESP), Faculdade de Odontologia de Araraquara, Departamento de Diagnóstico e Cirurgia, Divisão de Cirurgia Oral e Maxilofacial, Araraquara, SP, Brazil; d Universidade de Araraquara (Uniara), Faculdade de Medicina, Araraquara, SP, Brazil; e Universidade Estadual de Campinas (UNICAMP), Faculdade de Ciências Médicas, Departamento de Cirurgia, São Paulo, SP, Brasil

**Keywords:** Nasal cavity, Obstructive sleep apnea, Nasal obstruction

## Abstract

**Introduction:**

Obstructive sleep apnea is a consequence of upper airway collapse. Any obstructive sector in the upper airway can contribute to pharyngeal collapse. Obesity and obesity-related disorders play an important role in obstructive sleep apnea and its relationship with increased upper airway resistance.

**Objective:**

This study was designed to evaluate the relationship between obesity and properties of the nasal cavity in patients with obstructive sleep apnea.

**Methods:**

The study was conducted retrospectively by review of medical records of adult patients. The nasal obstruction symptom evaluation, NOSE instrument, was used to measure nasal obstruction. Sleep breathing disorders were evaluated by polysomnography exams. Nasal volume was obtained by computed tomography scans and volumetric reconstruction of nasal airway. Nasal anatomic alterations were assessed by nasal endoscopy.

**Results:**

Analysis of 83 patient records, among whom 54 were male and 29 females, found the mean body mass index of 28.69 kg/m^2^. Obese and non-obese groups were determined by using cut-off 30 kg/m^2^. In the comparison between groups, the obese group had a positive and significant correlation with apnea/hypopnea index (*p* = 0.02), NOSE instrument (*p* = 0.033) and inferior turbinate hypertrophy (*p* = 0.036), with odds ratio 1.983 (95% IC 1.048 − 3.753). nasal septum deviation (*p* = 0.126) and nasal airway volume evaluation (*p* = 0.177) showed no significant results.

**Conclusion:**

Obesity was significantly correlated with subjective nasal obstruction, NOSE scale, and inferior turbinate hypertrophy in patients with obstructive sleep apnea. There was no correlation with the nasal volume evaluation.

**Level of Evidence:**

3b - Individual case-control study.

## Introduction

The main pathophysiological site of obstructive sleep apnea (OSA) is the upper airway (UA).^1^ The American Academy of Sleep Medicine defines OSA as a recurrent upper airway collapse during sleep, resulting in a total (apnea) or partial (hypopnea) reduction of airflow.

Obesity is an isolated risk factor for OSA and its progression,^2^, ^3^, ^4^, ^5^ and obesity-related disorders play an important role in OSA; its relationship with increased upper airway resistance has been shown in various studies.^6^, ^7^, ^8^ Two-thirds of OSA patients are obese.^9^ A gain of 10% in baseline weight has been linked to a six fold-increased risk for OSA,^10^ and an increase of one standard deviation in BMI has been associated with a 4 fold increase in AHI.^11^ Metabolic dysregulation in subjects with OSA-Obesity, such as an increase in leptin levels, may lead to an increase in appetite and decrease in physical activity. Sleep deprivation and anxiety are also associated with this condition. This set of factors leads to a vicious circle that perpetuates this association.^4^

Patients with OSA-Obesity have been found to have fat deposition in the tissues surrounding the upper airway, increasing its collapsibility. Schwab et al., 2003, in a study using volumetric magnetic resonance imaging, found an increased fat deposition on the base of the tongue and lateral pharyngeal wall. These sites are more obstructive and therefore an important site of upper airway collapse in OSA subjects.^12^

To analyze UA stability, it is important to consider the external forces that compound the tissue pressure, whose main factor is obesity.^12^ Increased adipose tissue in the neck presses on the pharyngeal wall, causing more negative transmural pressure. The upper airway muscles of these patients have a primary myopathy, which makes them more susceptible to collapse because of an accumulation of muscle composed of the more fatigable fiber type II.^13^

The presence of a point of upper airway obstruction (UAO) increases intraluminal negative pressure in the section located after the point of obstruction, which triggers the narrowing of this segment. Patients with nasal obstruction are more exposed to the collapse of the upper airways by increasing negative pressure on the lumen of the pharynx.^14^ The nose contributes to half of total upper airway resistance.^15^

There are several methods to evaluate the nose and analyze how nasal alterations affect UA resistance. Endoscopic and computed tomography (CT) findings are important for the identification of obstructive sites. The nasal obstruction symptom evaluation scale (NOSE) is a validated subjective test for evaluating nasal symptoms.^16^ In this study the nasal volume was measured by CT volumetric reconstruction of the free spaces in the nasal cavity. Thus, the purpose this study was to evaluate whether there was any correlation between obesity and subjective/objective properties of the nasal cavity in patients with OSA.

## Methods

This study was approved by the ethical committee in research, under the number 47794715.3.0000.5416, and all procedures followed were in accordance with the ethical standards of the responsible committee on human experimentation (institutional and national) and with the Helsinki Declaration of 1975, as revised in 2008.

It was carried out retrospectively by reviewing medical records of adult patients from the Oral and Maxillofacial Surgery Clinic and Otorhinolaryngology Clinic. Patients were evaluated at a specific ambulatory clinic for patients with complaints and symptoms related to respiratory sleep disorders. The following data were obtained from the medical records: otorhinolaryngology (ENT) examination, upper airway endoscopy, anthropometric variables, Body Mass Index (BMI), baseline polysomnography, and Computed Tomography (CT) scans to define the nasal cavity volume.

The NOSE Instrument (Nasal Obstruction Symptom Evaluation) was used to grade nasal obstruction. The scale consisted of 5 questions (nasal congestion, nasal blockage, trouble with breathing, exercise and sleeping) receiving scores ranging from 0 to 4. These scores were added and multiplied by 5. Thus, the NOSE Instrument ranged from 0 to 100.^16^ This instrument is routinely applied to all patients with suspicion of OSA. All questionnaires were validated by the senior physician of the ambulatory clinic (MMR).

Upper airway endoscopy was performed to evaluate the anatomic alterations in the nose and pharynx. Possible findings included nasal septum deviations, inferior turbinate hypertrophy, tonsil hypertrophy and unstable sites of pharynx and larynx. Nasal Septum deviations (NSD) were considered when the septum blocked the fiberscope path and/or there was contact with the lateral wall of the nose. The size of the inferior turbinates we graded as follows: Grade 0 – no turbinates; Grade 1 − the inferior turbinates occupying <25% of nasal cavity; Grade 2 − the inferior turbinate occupying 25%−50% of nasal cavity; Grade 3 − the inferior turbinate occupying 50%−75% of the cavity; Grade 4 − the inferior turbinate occupying almost the nasal cavity and touching nasal septum.^17^, ^18^, ^19^ Inferior Turbinate Hypertrophy (ITH) was considered when the turbinate blocked the fiberscope path or Grades 3 − 4. All endoscopies were performed by means of a standardized protocol and validated by the senior physician of the ambulatory clinic. All procedures were recorded on video and described in a written report.

Sleep was assessed by polysomnography (PSG) type I, in an average period of six hours. The electrophysiological parameters evaluated during sleep were: electroencephalography (EEC), electrooculography (EOG), electromyography (EMG), electrocardiography (ECG), airflow (nasal and oral), respiratory effort (thoracic and abdominal), other body movements (by means of EMG), blood gases (oxygen saturation, carbon dioxide concentration) and body temperature. The technique was used as defined by the rules for scoring respiratory events in sleep from the American Academy of Sleep Medicine, 2012 manual.^17^ The severity of OSA was classified using apnea-hypopnea index as defined by AASM.^18^ A medical specialist in sleep calculated the patients’ apnea-hypopnea index (AHI), obtained by the sum of the apnea and hypopnea events divided by the hours of sleep. This index was used to classify the severity of OSA as follows: normal (AHI < 5 events/hour), mild OSA (AHI between 5 and 15 events/hour), moderate OSA (AHI between 15 and 30 events/hour) and severe OSA (AHI > 30 events/hour).

The CT images were not a routine at this ambulatory visit. The patients that had a CT scan in our file were included in this study. The CT were obtained by means of 128 channel tomography with the patients placed in the supine position. All subjects were instructed to not swallow during image acquisition. The sections were obtained in the axial plane and reconstructed in the coronal plane, from the anterior nasal spine to the anterior limit of the nasopharynx. All images were stored on a DVD for later analysis by specific software.

The tridimensional images of the CT scans were imported and reconstructed by using the Osirix v.7.0 32 bit software (OsiriX Foundation, Geneva, Switzerland) to define the nasal volume.^19^ The images were fixed in the Frankfurt plane, perpendicular to the horizontal plane, by image software. Weissheimer et al. (2012), has demonstrated that Osirix Software was validated for evaluating the upper airway volume.

Thus, images would be generated, corresponding to consecutive coronal sections of the region of interest, with spacing of 4 mm. A trained blinded observer conducted the evaluation process. The evaluation was repeated in 30% (minimum value to validate the standard error) of the sample, by the same evaluator, after a minimum period of 30 days to establish the method error. The values obtained in the reevaluation were similar to those of the first measures.

**Figure 1:**
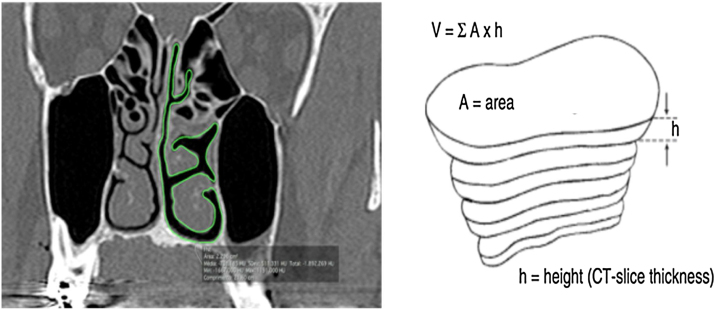
Coronal cut area delimitation and volume reconstruction. (A, CT coronal Cut; B, Reconstruction Technique) (From Rodrigues et al., 2017).^5^.

All measurements were performed in the coronal plane of CT slices with a thickness of 0.25 mm and 4 mm distance between slices. To determine the nasal airway volume, the area was measured in all the CT slices. The outline of the nasal airway in each slice was manually traced by means of the computer trackpad, considering only the free space of the nasal cavity; that is, turbinate and septum deviations were not included in the area calculation (Fig. 1). The software tool OsiriX calculated the area automatically. Then for each section of the CT, the area already given, a TIFF image was generated. This process was performed in both nasal cavities in the same slice in the coronal plane.

The Nasal Airway Volume (NAV) measured in each computed tomography slice was calculated by multiplying the area and height, which was equivalent to the distance between the coronal slices. The OsiriX software tool calculated the area of the missed slices spaced by 4 mm. The volume of the entire nose free airway was the sum of all volumes measured in each slice. NAV was the sum of volume obtained from both sides of the nasal cavity. The NAV obtained was similar to a pyramidal structure composed of the free airway space of the nose.

## Inclusion/exclusion criteria

Patients evaluated from December 2014 to December 2015, with OSA diagnosis, were included; both genders with age ranging between 18 and 70 years.

We excluded patients with the following conditions:

Morbid Obesity (BMI > 40), due to technical limitations on CT scan machine.

Craniofacial abnormalities (cranio-dysostosis, craniostenosis and meningomyelocele);

Nasal obstruction due to nasal polyps;

Presence of any craniofacial or airway tumor;

Laryngeal and pharyngeal paralysis;

Previous surgery on the UA.

Absence of CT or PSG records.

### Statistical analysis

Data were analyzed by statistical descriptive tests and frequency of results. The Kolgomorov-Smirnov Normality Test was used to establish the adequate test for variables. For the Nasal Volume and Nose Scale correlation analysis, the Mann-Whitney test was chosen. Chi-Square test was chosen for analysis of nominal variables. SAS System for Windows (Statistical Analysis System), version 9.3 software (SAS Institute Inc, 2002 − 2008, Cary, NY, USA) was used for the analysis.

## Results

Ninety patients were evaluated from December 2014 to December 2015. Seven patients were excluded from the sample because three had tomographic scans with poor definition of the limits determined in the methodology, and four had incomplete data necessary for the research protocol. Therefore, 83 patients were included in the study, 29 (34.9%) females and 54 (65.1%) males. The descriptive analysis of the main variables is shown in Table 1.

**Table 1:** Descriptive variables by Kolmogorov-Smirnov Test.

	BMI	NOSE scale	AHI	Nasal volume
**n**	83	83	83	83
**Mean ± Std. deviation**	28.70	39.52	27.94	17.25
	4.06	19.67	27.59	4.15
**Kolmogorov-Smirnov Z**	0.629	2.296	1.64	0.800
***p***	0.824	0.001	0.009	0.545

The above-mentioned test showed that the NOSE Scale and AHI did not have a normal distribution in this sample.

The sample variables were evaluated in gender groups. The analysis is shown in Table 2.

**Table 2:** Mean Comparison in Gender Groups.

	Gender	Mean	Std. deviation	*p*
**NOSE**	Male	40.95	20.60	0.935^a^
	Female	39.00	16.73	
**AIH**	Male	36.18	29.47	0.0001^a^
	Female	10.24	8.24	
**Nasal volume**	Male	17.97	4.35	0.155^b^
	Female	15.85	3.74	
**BMI**	Male	29.07	3.90	0.474^b^
	Female	27.97	4.05	

a Mann-Whitney test.

b Students*-t* test.

This data demonstrate that gender does not influence the nasal variables. The statistical significance was found only in AIH as literature preview. The subjects were divided into Obese and Non-Obese groups using a cut-off of 30 kg/m^2^ (Table 3).

**Table 3:** Group Statistics by Obesity Group.

	Obesity group	n	Mean	Std. deviation	Percent	*p*
**NOSE Scale**	Non-obese	56	37.11	18.16	−	0.033^a^
	Obese	27	44.52	21.99	−	
**AHI**	Non-Obese	56	22.47	23.59	−	0.020^a^
	Obese	27	39.28	32.02	−	
**Nasal volume**	Non-Obese	56	16.78	3.85	−	0.177^b^
	Obese	27	18.29	4.62	−	
**ITH**^c^	Non-obese	56	−	−	13/56 (23%)	0.036^e^
	Obese	27	−	−	13/27(48%)	
**NSD**^d^	Non-obese	56	−	−	29/56 (51%)	0.126^e^
	Obese	27	−	−	18/27(66%)	

a Mann-Whitney test.

b Student’s*-t* test.

c Inferior Turbinate Hypertrophy.

d Nasal Septum Deviation.

e Chi-Square Test.

The data above demonstrated that the obese group had a significant positive correlation with the NOSE Scale (*p* = 0.033) and AHI (*p* = 0.020). There was no difference in the Nasal Volume evaluation (*p*  = 0.177) between the groups. Cross tabulation between Inferior Turbinate Hypertrophy (ITH) and Obesity showed a significant correlation (*p* = 0.036). Odds ratio for ITH in obese group was 1.983 with 95% Confidence Interval of 1.048 − 3.753. There was no significant difference for NSD.

The ITH group had a significant positive correlation with the NOSE Scale (*p* = 0.041) and BMI (*p* = 0.044). There was no difference in the Nasal Volume evaluation (*p* = 0.198) and AHI (*p* = 0.051) between the groups (Table 4).

**Table 4:** Group Statistics by ITH Group.

	ITH	Mean	Std. deviation	*p*
**NOSE**	Yes	45.86	21.46	0.041^a^
	No	37.81	18.38	
**AIH**	Yes	33.93	31.42	0.051^a^
	No	23.10	24.65	
**Nasal volume**	Yes	17.09	5.08	0.198^b^
	No	17.36	3.98	
**BMI**	Yes	29.70	3.49	0.044^b^
	No	27.96	3.94	

a Mann-Whitney test.

b Student’s*-t* test.

## Discussion

Sleep apnea is an upper airway disease in which the pharynx is the main site affected. Nasal obstruction should be considered in the analysis of the balance between the opening and collapsing forces. Patients with nasal obstruction are more exposed to upper airway collapse by increasing negative pressure on the lumen of the pharynx.^14^ Studies have shown that nasal obstruction is a risk factor for OSA but there is no linear association between obstruction and severity of sleep-disordered breathing.^1^ Approximately 15% of patients with sleep-disordered breathing also have nasal obstruction.^20^

In this sample it was demonstrated that Endoscopic Nasal findings (NSD/ITH) are more important than NAV in appreciation of OSA patients. In the calculation of NAV the entire nasal cavity was considered, including the segment after a possible point of maximum obstruction, a gain of air flow that decreases intraluminal pressure in cranial segments of upper airway, leading a collapse of upper airway in the pharynx.^21^

The correlation between Nasal Obstruction and OSA is controversial. Haddad et al., 2013, evaluated nasal function with nasofibroscopy, nasal inspiratory peak flow and acoustic rhinometry in CPAP adherence. The results demonstrated that the majority of the nasal parameters evaluated in this study did not influence CPAP adherence.^21^

In our sample, obese subjects had more nasal symptoms, as shown by the NOSE Scale. Inferior Turbinate Hypertrophy was also correlated with obesity. Obese/OSA patients have 1.983 times greater chance of developing ITH. Subjects with ITH had higher NOSE scores, higher BMI and similar Nasal Volume when compared with non-ITH group. This result can demonstrate that ITH had a relationship with obesity independently of Nasal Volume and gender. Martinho et al., 2008, found similar results: 65.7% of OSA/Obese patients had ITH (*p* < 0.05).^22^ This correlation was not significant when NAV was evaluated. NAV is a variable that evaluates all the free airway space but does not evaluate the airflow in the nose. For example, in a patient with a nasal airway blocked by ITH, NAV will include the volume after and above the airway blockage; that is, a site with compromised airflow.

Gender did not influence the relationship between obesity and nasal function. There is no difference in NOSE and Nasal Volume scores. It’s expected that female subjects have a smaller nasal cavity, but this link was not observed in this sample. The only significant difference was shown in AIH that is reflected in the literature.^11^

Demir et al., 2015, found similar results, showing a statistically significant correlation between the increase in body mass and the increase in NOSE score, but they found no significant result when they evaluated nasal function using acoustic rhinometry; however, in their study they did not evaluate patients with OSA.^6^

Obesity and OSA are conditions linked to higher levels of inflammatory cytokines in nasal tissue, such as C-reactive protein, tumor necrosis factor alpha, interleukins (4, 13, 5, 8, 9), and granulocyte-macrophage colony-stimulating factor. OSA-Obese patients have more inflammatory markers in the tissue.^23^ The inferior turbinate is contractile and is the main site exposed to changes by inflammatory status of the nose. Inflammatory markers and edema resulting from respiratory trauma and snoring could explain the increased inferior turbinate findings in OSA-Obese subjects.^22^ Obesity also was pointed out as a cause of failure of treatment of the inferior turbinate conditions with radiofrequency.^24^

Obesity is an important condition that contributes to daytime Leg Fluid Accumulation (LFA), such as heart failure and end-stage renal disease.^25^, ^26^ Significant correlation has been shown between OSA and LFA. During recumbence the leg fluid was redistributed rostrally; approximately 260 mL of fluid was redistributed from the legs and was associated with a 1 cm increase in neck circumference, indicating neck fluid accumulation.^27^ These studies have suggested that alterations in upper airway mucosa could directly affect the inferior turbinate in OSA/obese patients by more fluid redistribution and more chronic inflammation in the nasal mucosa.

The main treatments of OSA in obese patients are CPAP therapy and bariatric surgery.^28^ The AHI Increases as Weight Increases.^29^ OSA is common in patients undergoing gastric bypass surgery. Bariatric surgery can reduce BMI and decrease fat pads surrounding the pharynx. Buchwald et al., 2004 in a meta-analysis showed that OSA was resolved in 85.7% of patients undergoing bariatric surgery.^30^

Nevertheless, nasal surgery appears to reliably augment CPAP compliance when nasal patency is the limiting issue.^31^ When evaluating OSA in obese patients it is important not to look for alterations in the pharynx alone. The Obese/OSA patients have more nasal symptoms. Our results demonstrated the importance of full evaluation of OSA patients. Nasal obstruction treatments cannot improve AHI but nasal steroids and nasal surgery can improve sleep and quality of life.^32^ The nasal examination is fundamental for appropriate CPAP adherence mainly in obese-OSA patients who, in our sample, had 1.983 times more risk of nasal obstruction due to ITH. This relationship can be explained by the increase in inflammatory markers, mucosa vibration and LFA redistribution in obese patients.^33^, ^34^

## Conclusion

In conclusion, obesity was correlated with subjective nasal obstruction (NOSE Scale) and inferior turbinate hypertrophy in patients with OSA. There was no correlation with the Nasal Volume evaluation. Thorough nasal evaluation is important in OSA patients to create a suitable treatment plan.

## Conflicts of interest

The authors declare no conflicts of interest.
